# The Role of Collectins and Galectins in Lung Innate Immune Defense

**DOI:** 10.3389/fimmu.2018.01998

**Published:** 2018-09-04

**Authors:** Cristina Casals, María A. Campanero-Rhodes, Belén García-Fojeda, Dolores Solís

**Affiliations:** ^1^Centro de Investigación Biomédica en Red de Enfermedades Respiratorias (CIBERES), Instituto de Salud Carlos III, Madrid, Spain; ^2^Departamento de Bioquímica y Biología Molecular, Universidad Complutense de Madrid, Madrid, Spain; ^3^Instituto de Química Física Rocasolano, CSIC, Madrid, Spain

**Keywords:** respiratory pathogens, infection, inflammation, surfactant proteins, alternatively activated macrophages, autophagy, tissue repair, lung homeostasis

## Abstract

Different families of endogenous lectins use complementary defense strategies against pathogens. They may recognize non-self glycans typically found on pathogens and/or host glycans. The collectin and galectin families are prominent examples of these two lectin categories. Collectins are C-type lectins that contain a carbohydrate recognition domain and a collagen-like domain. Members of this group include surfactant protein A (SP-A) and D (SP-D), secreted by the alveolar epithelium to the alveolar fluid. Lung collectins bind to several microorganisms, which results in pathogen aggregation and/or killing, and enhances phagocytosis of pathogens by alveolar macrophages. Moreover, SP-A and SP-D influence macrophage responses, contributing to resolution of inflammation, and SP-A is essential for tissue-repair functions of macrophages. Galectins also function by interacting directly with pathogens or by modulating the immune system in response to the infection. Direct binding may result in enhanced or impaired infection of target cells, or can have microbicidal effects. Immunomodulatory effects of galectins include recruitment of immune cells to the site of infection, promotion of neutrophil function, and stimulation of the bactericidal activity of infected macrophages. Moreover, intracellular galectins can serve as danger receptors, promoting autophagy of the invading pathogen. This review will focus on the role of collectins and galectins in pathogen clearance and immune response activation in infectious diseases of the respiratory system.

## Introduction

Host defense in the lung is exceptionally, if not uniquely, challenging. The alveolar boundary is clearly the most vulnerable body interface. There are at least three important differences among the alveolar boundary and the upper respiratory tract, gut, and skin interfaces. First, the surface area to be defended is greater in the alveolar boundary (90 m^2^) than in the gut (10 m^2^) or skin (2 m^2^) (1). Second, compared with the skin, gut, and upper respiratory tract, the bacterial biomass in the alveoli of healthy lungs is low (2). Third, there are physical barriers or harsh chemical environments in the skin (cornified epithelial layers) and gut (regular secretion of bile, which acts as an antiseptic detergent) but not in the delicate alveolar space. In addition, there is higher risk of pathogen dissemination at the alveolar boundary than at any other environmental boundary, since only two cell layers (the alveolar epithelium and the capillary endothelium) separate the invader from the bloodstream in order to facilitate gas exchange.

The innate immune system in the alveolar space is made up of a cellular arm [mainly alveolar macrophages (aMΦ) (1), but also epithelial (AEC), dendritic, and T cells (1, 3, 4)] and a humoral arm composed of antimicrobial proteins and peptides present in the alveolar fluid such as surfactant protein A (SP-A) and D (SP-D), lactoferrin, lysozyme, fibronectin, immunoglobulins, complement components, defensins, and cathelicidins, among others (5, 6). In this review we focus on the role of lung soluble collagenous C-type lectins (SP-A and SP-D) and galectins. SP-A and SP-D are principally secreted to the alveolar fluid by type II AECs and to the airway lumen by Club cells and submucosal cells (7). They are also detected in the trachea (8) and nasal mucosa (9), where they provide immune protection. Galectins are expressed in the lung by innate immune cells and epithelial cells. Galectins are present in the cytoplasm and nucleus, as well as extracellular space, although galectins lack a typical secretion signal peptide. They are secreted by direct translocation across the plasma membrane or through release in extracellular vesicles (10). Thus, they can function both inside and outside cells. The review describes biochemical and structural aspects of lung collectins and their role in antimicrobial immunity and alveolar immune homeostasis, and the involvement of galectins in the response to respiratory infectious diseases, including expression, binding to pathogens, modulatory effects on immune cells, and intracellular functions.

## Collectins

### Biochemical and structural aspects

**Figure 1:**
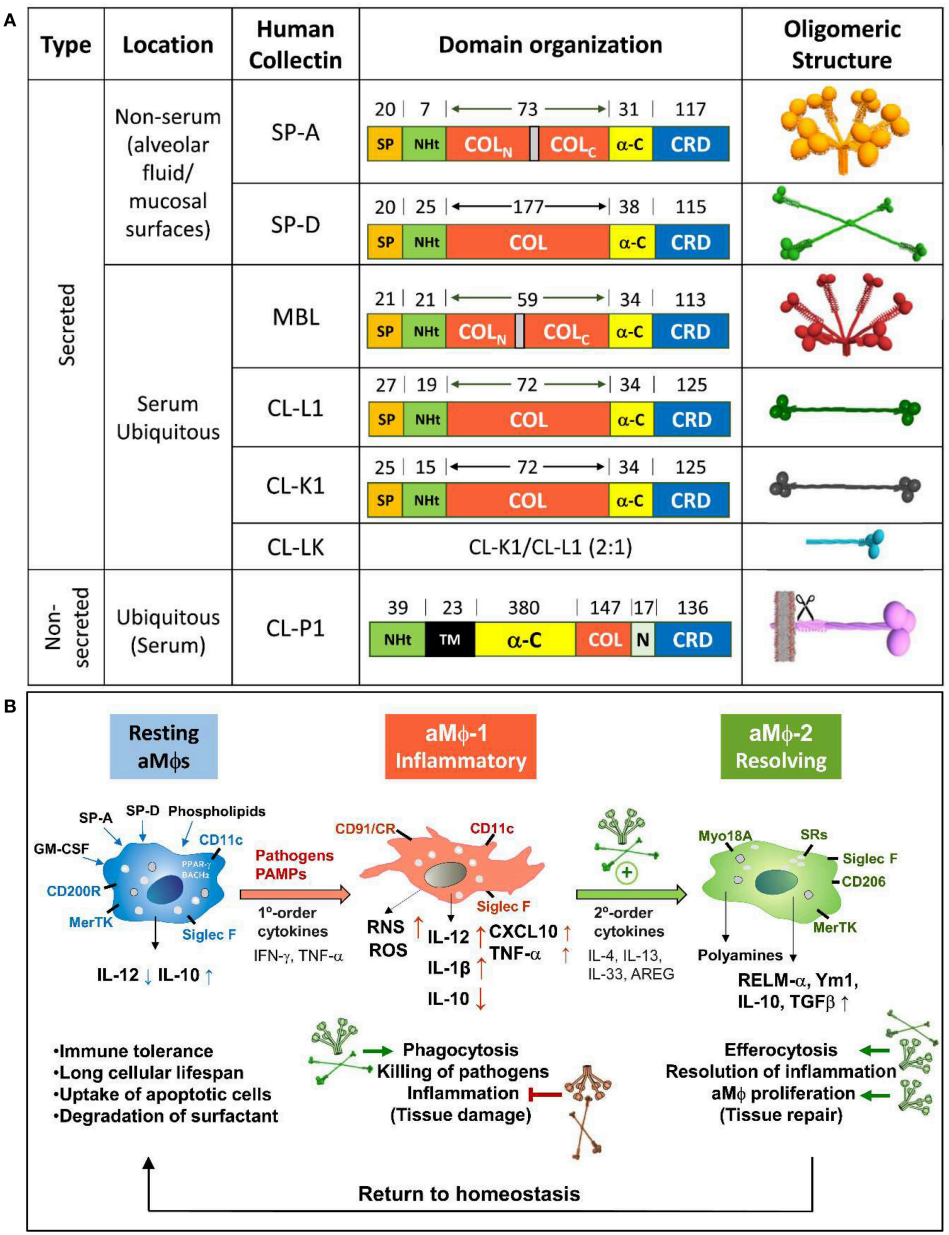
**(A)** Structural analysis of human collectins. Domain organization of human collectin polypeptide chains and the number of amino acids covering each domain are shown. Interruptions in the collagen domain of SP-A and MBL are indicated. Three-dimensional models of collectin oligomers are also shown. Trimers of collectins are each built up by the association of three polypeptide chains, the collagen regions of which intertwine to form a collagen triple helix. Whereas all other collectins are soluble, CL-P1 is a transmembrane protein orientated with its N-terminal toward the cytosol. CL-P1 may be regarded as both a collectin and a scavenger receptor. The scissors symbol means the shedding of a soluble form of CL-P1 by a hitherto unknown mechanism, which results in the presence of soluble CL-P1 in the circulation. The molecules are not drawn to scale. SP, signal peptide; NHt, N-terminal domain; COL, collagen-like domain; α-C, α-helical coiled-coil domain; CRD, carbohydrate recognition domain; TM, transmembrane domain. **(B)** Role of lung collectins on sequential type 1 and type 2 immune responses following respiratory infection. Respiratory pathogens are detected by AECs and aMΦs, initiating an innate immune response to clear localized infections. The type 1 response is essential in controlling infection but also induces tissue damage. Stimulated tissue-resident lymphoid cells and AECs release appropriate second-order cytokines that initiate a two-tiered response. The type 2 response, amplified by lung collectins (14), modulates aMΦs toward an anti-inflammatory resolving phenotype involved in lung repair. The role of lung collectins in these homeostatic changes is shown by small green or red arrows, which mean SP-A/D-mediated activation or inhibition, respectively.

Collectins or collagenous C-type lectins are a family of proteins that contain a Ca^2+^-dependent carbohydrate recognition domain (CRD) contiguous to a collagen-like triple helical domain. In humans, members of this group include SP-A and SP-D, secreted by the alveolar epithelium, nonciliated bronchiolar cells and other mucosal surfaces exposed to the external environment (7, 8, 11), mannan-binding lectin (MBL) secreted by hepatocytes to serum, and the recently discovered CL-L1, CL-K1, and CL-P1, present in the serum and several tissues (12, 13) (Figure 1A). Collectins are well-conserved oligomeric proteins, assembled in trimers or multiples of three subunits due to their collagen domains. The primary structure of each subunit consists of an N-terminal segment containing cysteine residues involved in oligomerization followed by a collagen-like region, an alpha helical coiled neck region, and a globular CRD with a calcium ion at the lectin site (Figure 1A). Lung collectins are modified after translation (cleavage of the signal peptide, proline hydroxylation, and N-linked glycosylation) (7, 15) and intracellularly assembled into oligomeric structures that, in the case of SP-A, resemble a flower bouquet of six trimers, while the assembly of SP-D resembles a cruciform of four trimers (Figure 1A). Supratrimeric oligomerization of lung collectins appears to be needed for many of their functions (16, 17) since it facilitates multivalent binding and increases the functional affinity of the globular domain for their ligands.

The CRDs of lung collectins bind to mannose and mannose-rich microbial glycoconjugates, such as yeast mannans and mycobacterial lipoarabinomannan. Besides, lung collectins recognize a wide variety of carbohydrates present in the surface of several microorganisms, including glucose, fucose, N-acetylglucosamine, and N-acetylmannosamine. Their globular domains recognize not only carbohydrates but also a broader repertoire of ligands, including proteins, nucleic acids, and lipids (7, 18, 19). Despite their similar CRDs, SP-A and SP-D show significant differences in ligand preferences, with SP-A ligands generally being more amphipathic (19, 20), and SP-D ligands richer in carbohydrates (20). These different preferences likely serve to extend the range of innate immune surveillance in the lung.

The collagen-like domains of collectins not only function as scaffolding that amplifies the ligand binding activities of globular domains but also are responsible for collectin binding to receptors in immune cells. Such receptors are involved in phagocytosis and clearance of microorganisms (7, 8, 12, 13) and biological/abiotic particles from the pulmonary environment (21–23), and in efferocytosis of dead cells (apoptotic/necrotic) (24, 25). Collagen-dependent functions of collectins are shared with other secreted defense collagens (C1q, ficolins, and adiponectin) (26). This group of proteins has a dual capacity to promote pathogen elimination and control inflammation (27–30). Moreover, they seem to activate molecular and cellular mechanisms that force a return to homeostasis (14).

### Antimicrobial immunity

SP-A and/or SP-D recognize a wide range of respiratory pathogens, including influenza A virus, respiratory syncytial virus, *Mycobacterium tuberculosis, Aspergillus fumigatus, Pseudomonas aeruginosa, Haemophilus influenzae* [see (8, 31) for reviews], and the parasitic helminth *Nippostrongylus brasiliensis* (32). SP-A- or SP-D-deficient mice show decreased microbe clearance and increased tissue markers of inflammation (14, 30–33), suggesting lung collectins' protective role in lung immune defense.

Lung collectins enhance the clearance of pathogens by four different mechanisms: (i) By aggregating pathogens to which they bind, which hinders their entry into epithelial cells and facilitates their removal, either by mucociliary clearance or by phagocytosis by aMΦs and recruited neutrophils (7, 8, 30, 31, 34). (ii) By binding to neutrophil extracellular traps (NET)-DNA and to bacteria simultaneously, thereby promoting bacterial trapping by the NETs (35). (iii) By enhancing phagocytosis of IgG-opsonized particles (36) and complement-coated particles (36, 37). (iv) By up-regulating expression of cell-surface receptors involved in microbial recognition, such as mannose receptor (38) and scavenger receptor SR-AI/II (39).

Data supporting direct antimicrobial activity of SP-A and SP-D are sparse (8, 31). Most respiratory pathogenic bacteria and fungi are resistant to SP-A and SP-D (8, 34, 40, 41). However, it is possible that cooperative interactions of lung collectins with other lung antimicrobial peptides enhance the microbicidal defense of the lungs. In this regard, we recently discovered synergic action between SP-A and SP-B^N^, a secreted anionic antimicrobial peptide derived from SP-B proprotein. Interaction between SP-A and SP-B^N^ confers new antimicrobial properties, including the ability to bind, kill, and enhance phagocytosis of pathogenic *K. pneumoniae* K2 that is otherwise resistant to either protein alone (34). Moreover, therapeutic treatment with SP-A and SP-B^N^ protects against *K. pneumoniae* K2 infection *in vivo* due to SP-A/SP-B^N^ capability to both kill bacteria and modulate host inflammatory response (34). Yet a promising field to explore is the interaction of lung collectins with other lung antimicrobial peptides and antibiotics and the potential relevance of these interactions in innate host defense in the lung.

### Alveolar immune homeostasis

The niche in which alveolar macrophages exist, rich in surfactant lipids, SP-A, and SP-D (42), has a considerable influence on many aspects of aMΦ phenotype (1, 43). Alveolar MΦs function as sentinels of a healthy state, promoting immune tolerance to innocuous antigens. During an infection, aMΦs recognize alarm signals such as IFN-γ and PAMPs, initiating proinflammatory responses and pathogen clearance (MΦ-1 phenotype) (Figure 1B), and collectins promote phagocytosis of pathogens by binding to the CD91/calreticulin receptor on aMΦs (44). However, host defense requires a balance between decreasing microbial burden and restricting tissue damage caused directly by pathogens or indirectly by the immune response (45). In this vein, lung collectins influence aMΦ responses to limit inflammation. First, they block the binding of TLR ligands to their receptors by direct interaction with TLR4, TLR2, the TLR co-receptor MD2, and CD14 (17, 30, 46) or by binding to TLR4/CD14 ligands (47, 48), acting as LPS scavengers *in vivo* (49). Second, they modify aMΦ response to TLR ligands by modulating signaling cascades. For example, SP-A and SP-D bind to SIRPα through their globular heads to initiate an SHP-1-dependent signaling pathway that blocks proinflammatory mediator production (44). In addition, SP-A increases the expression of negative regulators of TLR-signaling, such as IRAK-M (50) and β-arrestin 2 (51), and inhibits activation of NFκB, ERK, p38, and Akt in aMΦs (52, 53). Third, they reduce the production of reactive oxygen intermediates (54, 55) and for SP-D, this effect is mediated through its binding to the inhibitory receptor LAIR-1 (56). Fourth, SP-A limits inflammation by binding to IFN-γ, suppressing IFN-γ interaction with its receptor IFN-γR1 (57).

Besides limiting inflammation, lung collectins activate different mechanisms that contribute to disease resolution. After proinflammatory type 1 responses against invading pathogens, repair-associated type 2 responses must be initiated (58, 59). The tissue repair response is classically associated with the production of IL-4/IL-13 cytokines and the induction of alternatively activated macrophages (MΦ-2 phenotype) (Figure 1B). We recently found that defense collagens (SP-A and C1q) enhance IL-4/13-dependent alternative activation, proliferation, and tissue-repair functions of macrophages through binding to the myosin 18A receptor by their collagen domains (14). Loss of function studies using SP-A- and C1q-deficient mice demonstrated that SP-A and C1q are necessary to promote tissue repair during infection with the parasite *N. brasiliensis* and the Gram positive bacterium *Listeria monocytogenes*, respectively (14). SP-D also seems to be an important modulator of protective IL-4/13-induced aMΦ responses against *N. brasiliensis* (32). Interestingly, IL-4Rα signaling requires concomitant recognition of apoptotic cells to induce the tissue repair program in macrophages (60), suggesting that tissue repair is restricted to the damaged site. SP-A, SP-D, and C1q assist the recognition and clearance of apoptotic neutrophils by macrophages (24, 25, 61, 62), a mechanism that differs from classical phagocytosis and that leads to the production of anti-inflammatory cytokines (IL-10 and TGFβ) (63), contributing to host tolerance during lung infection.

In conclusion, pulmonary collectins provide immune protection against respiratory pathogens, promoting pathogen clearance, limiting inflammation, and activating molecular and cellular mechanisms that help to restore homeostasis. Much of what we know about the protective role of SP-A and SP-D has arisen from studies using SP-A– or SP-D–deficient mice in murine models of respiratory infections (14, 31–33) and other respiratory diseases (30, 31, 64).

## Galectins

Galectins are a family of lectins sharing a CRD with β-sandwich fold and β-galactoside-binding ability (65, 66). Nevertheless, the glycan-binding preferences of different galectins may differ significantly, leading to functional divergences. To date, 16 mammalian galectins have been described, of which galectins 5, 6 (both found in rodents), 11, and 15 (found in ruminants) are not present in humans (Figure S1). Based on their structural organization (Figure S1), galectins are classified as proto type, composed of one or two identical CRDs forming non-covalent homodimers (e.g., Gal-1), chimera type, composed of one CRD linked to a non-lectin N-terminal region (Gal-3), and tandem-repeat type, containing two different CRDs covalently connected by a linker peptide (e.g., Gal-8 and -9). Galectins are widely expressed in epithelial and immune cells, and participate in different biological phenomena, including inflammation and immunity (67, 68).

### The expression of galectins is altered in respiratory infections

An archetypal example is the accumulation of Gal-3 in the alveolar space of *Streptococcus pneumoniae*-infected mice (69). Gal-3 release also increases in the lungs of mice lethally infected with *Francisella novicida* (70). In patients infected with *M. tuberculosis*, the plasma levels of Gal-9 are significantly increased (71). However, Gal-9 expression in macrophages generated in the presence of *M. tuberculosis* lipoarabinomannan is down-regulated, favoring bacterial intracellular growth (72).

The expression of galectins is also affected by respiratory viruses. As an example, Gal-1 is up-regulated in the lungs of influenza virus-infected mice, in correlation with the viral load (73). Interestingly, Gal-1 is differentially expressed in bronchoepithelial cells infected with 2009 A or seasonal H1N1 influenza virus, revealing strain-specific responses (74). Moreover, patients carrying genetic variants associated with higher Gal-1 expression are less susceptible to infection by avian influenza A (75).

Gal-3 levels in serum and lungs are augmented in infections by the fungus *Cryptococcus neoformans* (76), while plasma levels of Gal-9 are higher in severe infections by the parasite *Plasmodium falciparum* (77), and mRNA levels of Gal-9 in the lungs of *P. berghei*-infected mice are also increased (78).

Thus, the expression of galectins is altered in bacterial, viral, fungal, and parasitic respiratory infections, conceivably correlating with galectin-mediated defense mechanisms.

### Galectins bind to different respiratory pathogens

**Figure 2:**
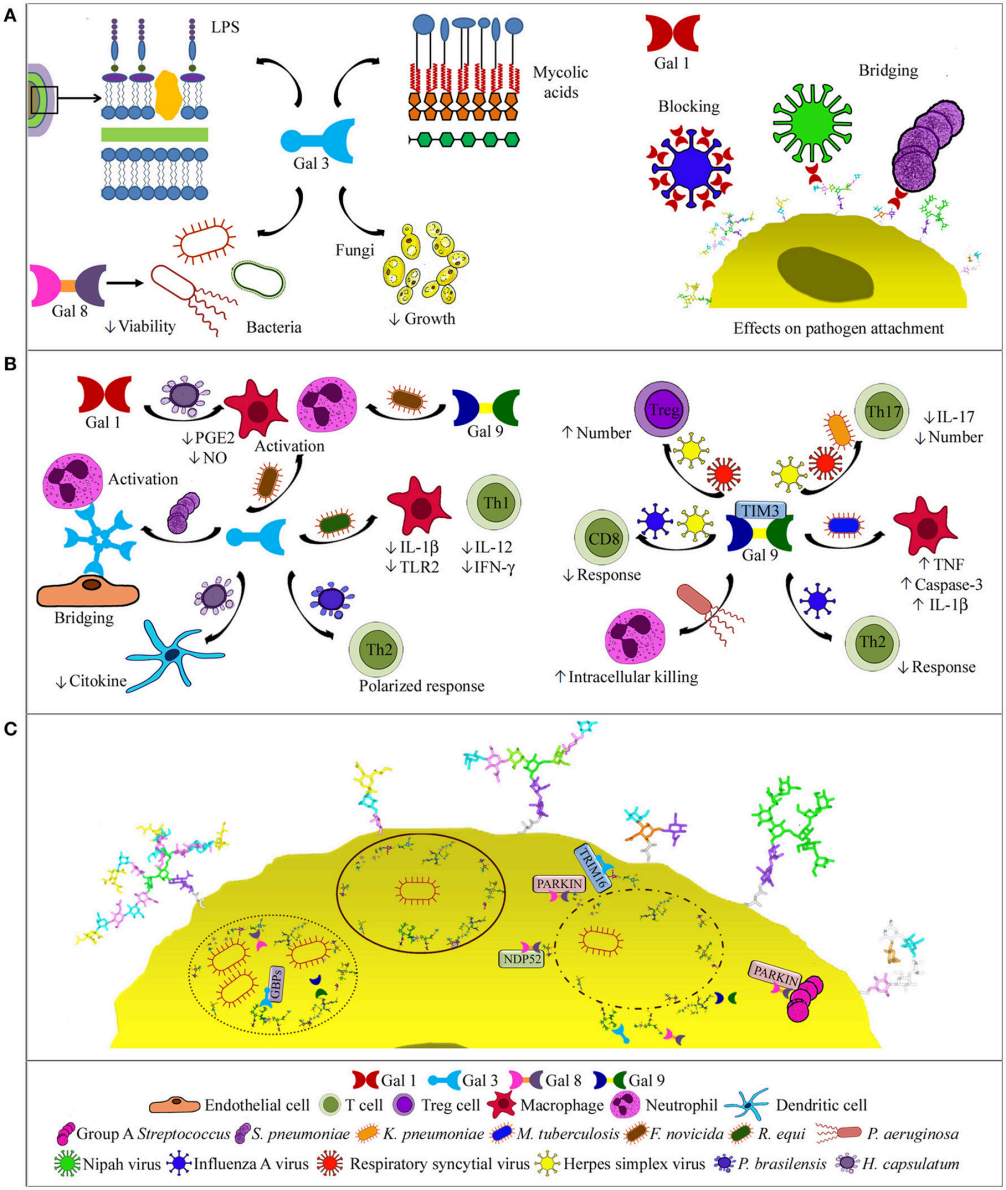
Galectin activities in respiratory infections. **(A)** Binding to pathogens. Gal-3, the only chimera-type galectin described to date, binds bacterial mycolic acids, lipopolysaccharides, and cells, and also *C. neoformans* cells with antifungal effects. Gal-8 binds NTHi, decreasing bacterial viability (left side). Gal-1 binding to influenza virus blocks infection, while binding to NiV and *S. pneumoniae* bridges pathogen and host glycans (right side). **(B)** Effects on immune cells. Oligomerized Gal-3 can bridge neutrophils to endothelial cells. Depending on the pathogen, Gal-3 drives a Th2-polarized response, decreases macrophage and Th1 cell responses, or activates macrophages and/or neutrophils, similarly to Gal-9. In histoplasmosis, Gal-3 decreases cytokine production by dendritic cells, while Gal-1 modulates PGE2 and NO levels (left). Via TIM-3 binding, Gal-9 may promote bacterial killing by neutrophils or macrophages, decrease humoral and CD8+ cell responses or Th17 cells and IL-17 levels, and increase Treg cells (right). **(C)** Intracellular functions. Gal-3, -8, and -9 bind to host glycans in the luminal side of lysed phagosomes or permeable replicative vacuoles, and contribute to the autophagic response by recruiting NDP52, parkin, GBPs, or TRIM-16. Gal-8 also recruits parkin to group A *Streptococcus*.

Gal-3 binds mycolic acids (Figure 2A), the major constituents of mycobacterial cell wall, and could participate in their interaction with host cells (79). Gal-3 also binds lipopolysaccharides from different bacteria, including *K. pneumoniae* (80) and *P. aeruginosa* (81). Moreover, Gal-3 and Gal-8 bind to *K. pneumoniae* O1 cells and decrease bacterial viability, and the same occurs for Gal-8 and strain 2019 of non-typeable *H. influenzae* (NTHi) (82). Recently, binding of Gal-8 to other six different NTHi clinical isolates was detected (83), suggesting that this could be a general trait.

At the initial phase of infection by Nipah virus (NiV), Gal-1 bridges glycans of the envelope glycoprotein F (NiV-F) with those of host cells, thereby enhancing virus attachment. However, Gal-1 secreted in response to infection reduces NiV-F-mediated syncytia formation and production of progeny virus (84–86). This is a remarkable example of opposing effects of the same galectin on infection by a given pathogen. Moreover, Gal-1 binds to envelope glycoproteins of influenza virus, impairing infection. Cells treated with Gal-1 generate lower viral yields, and treatment of infected mice reduces viral load and lung inflammation (73). Conversely, Gal-1 could account for the increased susceptibility of influenza patients to subsequent infection with pneumococcus. Influenza infection results in desialylation of epithelial cell glycans and exposure of galactosyl moieties that serve as galectin ligands. As Gal-1 also binds to *S. pneumoniae*, it crosslinks the bacteria to the airway epithelial surface, enhancing pneumococcal adhesion (87).

As final example, Gal-3 binds to *C. neoformans* cells and delays fungal growth. Moreover, it exerts a lytic effect on fungal extracellular vesicles (76). Thus, Gal-3 has direct anti-*C. neoformans* effects.

### Galectins modulate the immune response to infection

Accumulation of Gal-3 in the alveoli of pnemococcus-infected mice correlates with neutrophil extravasation (69). Consistently, less neutrophils are recruited in Gal-3^−/−^ mice, which develop more severe pneumonia, and treatment with Gal-3 reduces the severity of infection (88, 89). Gal-3 bridges neutrophils to endothelial cells and activate neutrophils (Figure 2B), augmenting pneumococcus phagocytosis and delaying apoptosis. These effects are disabled by *Staphylococcus aureus* via degradation of Gal-3 with a bacterial protease (90). In contrast, Gal-3 deficiency confers resistance to *Rhodococcus equi* (91). Thus, Gal-3^−/−^ mice exhibit higher bacteria lethal doses and production of IL-12 and IFN-γ. Moreover, Gal-3^−/−^ macrophages show decreased bacterial replication and survival, and enhanced production of IL-1β and TLR2. Gal-3^−/−^ mice lethally infected with *F. novicida*, however, show significantly reduced inflammatory response and leukocyte infiltration in the lungs, in parallel to improved lung architecture and survival (70), and the same is observed for Gal-9^−/−^ mice (92). Thus, Gal-3 and Gal-9 function as proinflammatory alarmins in *F. novicida* infection.

Gal-9 also modulates the immune response through binding to TIM-3 receptor on neutrophils, macrophages and lymphocytes. For example, *P. aeruginosa* opsonization with Gal-9 enhances neutrophil-mediated killing via TIM-3 interactions inducing intracellular Ca^2+^ mobilization, neutrophil degranulation, and NADPH oxidase activity (93). Binding of Gal-9 expressed by *M. tuberculosis*-infected macrophages to TIM-3 also leads to restriction of intracellular bacterial growth through secretion of IL-1β, upregulation of TNF, and activation of caspase-3 (94–96). In contrast, Gal-9–TIM-3 binding decreases the levels of IL-17 in serum of mice infected with *K. pneumoniae*, resulting in reduced bacterial clearance (97).

Gal-9 binding to TIM-3 receptor on T lymphocytes decreases the immune response against viral infections. Influenza A virus-infected Gal-9^−/−^ mice generate stronger humoral and CD8+ T-cell responses and cleared virus more rapidly than Gal-9^+/+^ mice. Accordingly, selective blocking of the Gal-9–TIM-3 interaction in Gal-9^+/+^ mice boosts the immune response (98). On the other hand, Gal-9 administered to mice infected with respiratory syncytial virus decreases the severity of lung pathology by increasing Tregs number and reducing the number of Th17 cells, IL-17 levels, and CD8+ T cell apoptosis (99). Similarly, Gal-9 injection into herpes simplex virus-infected mice increases Tregs number and decreases the levels of pro-inflammatory cytokines, improving the symptoms of inflammation (100), while intraperitoneal infusion of lactose, which prevents Gal-9 binding to TIM-3, reduces Treg function and augments CD8+ T cell responses (101).

In respiratory fungal infections, Gal-1 and Gal-3 play differential roles. Gal-1 modulates prostaglandin E2 and nitric oxide levels in *H. capsulatum* infection, contributing to phagocyte responses and thus exerting a protective effect (102). In contrast, Gal-3^−/−^ mice clear *H. capsulatum* infection more efficiently than Gal-3^+/+^ mice, likely due to a negative regulatory role of Gal-3 on cytokine production by dendritic cells (103). However, Gal-3^−/−^ mice are more susceptible to *P. brasiliensis* infection and present a Th2-polarized immune response, clearly showing that Gal-3 effects depend on the particular pathogen (104).

### Intracellular activities of galectins

After internalization into host cells, many bacteria lyse the phagosome and escape to the cytosol for establishing a replicative niche (Figure 2C). Galectins 3, 8, and 9 bind to damaged vacuoles that expose host glycans in the luminal side of the phagosome membrane (105, 106). Moreover, Gal-8 recruits the autophagy NDP52 receptor, activating phagosome degradation (106). Gal-8 also binds parkin, which targets damaged vesicles and bacteria for ubiquitination. Interestingly, Gal-3 diminishes the recruitment of Gal-8 and parkin to group A *Streptococcus*, which does not replicate in endothelial cells and organs of Gal-3^−/−^ mice (107). Gal-8 also targets for degradation damaged endosomes in picornavirus and adenovirus infections (108, 109).

Other bacteria replicate within the phagosomes, as e.g., *Coxiella burnetii*. Yet, galectins 3, 8, and 9 accumulate in the luminal side of the vacuole membrane, revealing membrane permeability (110). Gal-3 and Gal-8 are also detected in replicative vacuoles of *Legionella pneumophila* (111) and mediate delivery of guanylate binding proteins, a family of antimicrobial GTPases induced by IFN-γ (112). Moreover, Gal-3 binds TRIM-16, further contributing to organizing the autophagic response. The Gal-3-TRIM-16 system operates in macrophages infected with *M. tuberculosis* strains causing phagosome damage, and is required for bacteria translocation to lysosomes. Accordingly, Gal-3 protects mice in acute and chronic *M. tuberculosis* infection (113, 114).

Summarizing, galectins play diverse roles in respiratory infections with sometimes disparate effects, which may benefit the host or the pathogen, depending on the specific galectin, pathogen, and host context.

## Concluding remarks

This review touched briefly on the important role of collectins and galectins in pathogen clearance and immune response activation. Lung collectins are critical in mediating a variety of immune and physiological responses during health and disease. Galectins also mediate effective antimicrobial and immunoregulatory activities but, if activated inappropriately, can act as potent inducers of immunopathology. It remains to be determined whether collectins and galectins can interact with each other and whether such collaborations harness a beneficial immune response to pathogens. A more complete understanding of the host factors that control microbial colonization will lead to improved therapies for respiratory infections.

## Author contributions

CC, MC-R, BG-F, and DS contributed equally to the writing of this review article. CC and DS are co-corresponding authors.

### Conflict of interest statement

The authors declare that the research was conducted in the absence of any commercial or financial relationships that could be construed as a potential conflict of interest.
