# Supplementary material for: The Role of Collectins and Galectins in Lung Innate Immune Defense
Source: Front Immunol. 2018 Sep 4;9:1998. doi: 10.3389/fimmu.2018.01998 (PMC6131309; doi:10.3389/fimmu.2018.01998)
Supplement: Supplementary file 1 [file Presentation_1.pdf]

Supplementary Material

The role of collectins and galectins in lung innate immune defense

C. Casals[1,2]*, M.A. Campanero-Rhodes[1,3], B. García-Fojeda[1,2], D. Solís[1,3]*

[1]Centro de Investigación Biomédica en Red de Enfermedades Respiratorias (CIBERES), Instituto de Salud Carlos III, 28029-Madrid, Spain
[2]Departamento de Bioquímica y Biología Molecular, Universidad Complutense de Madrid, 28040 Madrid, Spain
[3]Instituto de Química Física Rocasolano, CSIC, 28006-Madrid, Spain

*Correspondence: Cristina Casals (ccasalsc@ucm.es) and Dolores Solís (d.solis@iqfr.csic.es)

A GALECTIN STRUCTURAL TYPES

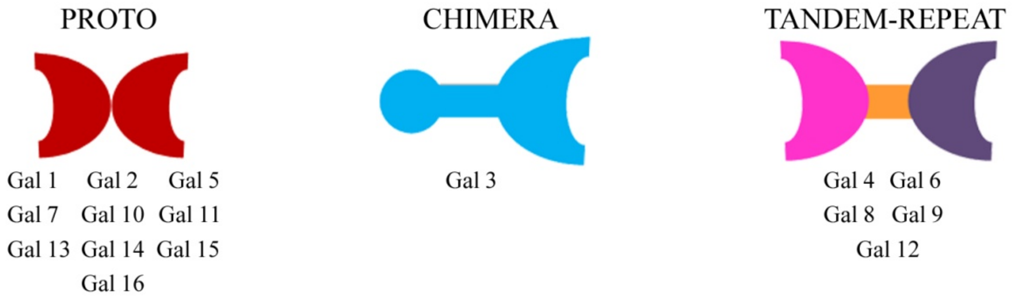

B MULTIPLE SEQUENCE ALIGNMENT OF HUMAN GALECTINS

```
HGAL1      -----------MA--CGLVASNLNLKPGECLRVRGEVAP---DAKSFVLNLGKDS-----
HGAL2      -----------MT--GELEVKNMDMKPGSTLKITGSIAD---GTDGFVINLGQGT-----
HGAL3      PATGPYGAPAGPLIVPYNLPLPGGVVPRMLITILGTVKP---NANRIALDFQRG------
HGAL4-ND   ---APGYQPTYNPTLPYYQPIPGGLNVGMSVYIQGVASE---HMKRFFVNFVVGQ--DPG
HGAL4-CD   ----------------YFGRLQGGLTARRTIIIKGYVPP---TGKSFAINFKVGS----S
HGAL7      -----------MSNVPHKSSLPEGIRPGTVLRIRGLVPP---NASRFHVNLLCGE--EQG
HGAL8-ND   ---NNLQNIIYNPVIPFVGTIPDQLDPGTLIVIRGHVPS---DADRFQVDLQNGSSMKPR
HGAL8-CD   ----------------FAARLNTPMGPGRTVVVKGEVNA---NAKSFNVDLLAGK----S
HGAL9-ND   -----SQAPYLSPAVPFSGTIQGGLQDGLQITVNGTVLSS--SGTRFAVNFQTG---FSG
HGAL9-CD   ----------------FITTILGGLYPSKSILLSGTVLP---SAQRFHINLCSG------
HGAL10     -----------MSLLPVPYTEAASLSTGSTVTIKGRPLACFLNEPYLQVDFHTE--MKEE
HGAL12-ND  PDSFILQPPVFHPVVPYVTTIFGGLHAGKMVMLQGVVPL---DAHRFQVDFQCGCSLCPR
HGAL12-CD  ----------------CSHALPQGLSPGQVIIVRGLVLQ---EPKHFTVSLRDQA-----
HGAL13     -----------MSSLPVPYKLPVSLSVGSCVIIKGTPIHSFINDPQLQVDFYTD--MDED
HAGL14     -----------MSSLPVPYTLPVSLPVGSCVIITGTPILTFVKDPQLEVNFYTG--MDED
HGAL16     -----------MSFLTVPYKLPVSLSVGSCVIIKGTLIDSSINEPQLQVDFYTE--MNED

HGAL1      NNLCLHFNPRFNAHGDANTIVCNSKDGGAWGTEQRE--AVFPFQPGSVAEVCITFDQANL
HGAL2      DKLNLHFNPRFSE----STIVCNSLDGSNWGQEQRE--DHLCFSPGSEVKFTVTFESDKF
HGAL3      NDVAFHFNPRFNENN-RRVIVCNTKLDNNWGREERQ--SVFPFESGKPFKIQVLVEPDHF
HGAL4-ND   SDVAFHFNPRFDGW---DKVVFNTIQGGKWGSEERK--RSMPFKKGAAFELVFIVLAEHY
HGAL4-CD   GDIALHINPRMGNGT----VVRNSLLNGSWGSEEKKI-THNPFGPGQFFDLSIRCGLDRF
HGAL7      SDAALHFNPRLDTS----EVVFNSKEQGSWGREERG--PGVPFQRGQPFEVLIIASDDGF
HGAL8-ND   ADVAFHFNPRFKRA---GCIVCNTLINEKWGREEIT--YDTPFKREKSFEIVIMVLKDKF
HGAL8-CD   KDIALHLNPRLNIKA----FVRNSFLQESWGEEERNI-TSFPFSPGMYFEMIIYCDVREF
HGAL9-ND   NDIAFHFNPRFEDG---GYVVCNTRQNGSWGPEERK--THMPFQKGMPFDLCFLVQSSDF
HGAL9-CD   NHIAFHLNPRFDENA----VVRNTQIDNSWGSEERSLPRKMPFVRGQSFSVWILCEAHCL
HGAL10     SDIVFHFQVCFGRR-----VVMNSREYGAWKQQVES--KNMPFQDGQEFELSISVLPDKY
HGAL12-ND  PDIAFHFNPRFHTTK--PHVICNTLHGGRWQREARW--PHLALRRGSSFLIILFLFGNEEV
HGAL12-CD  AHAPVTLRASFADRT--------LAWISRWGQKKLI-SAPFLFYPQRFFEVLLLFQEGGL
HGAL13     SDIAFRFRVHFGNH-----VVMNRREFGIWMLEETT--DYVPFEDGKQFELCIYVHYNEY
HAGL14     SDIAFQFRLHFGHP-----AIMNSCVFGIWRYEEKC--YYLPFEDGKPFELCIYVRHKEY
HGAL16     SEIAFHLRVHLGRR-----VVMNSREFGIWMLEENL--HYVPFEDGKPFDLRIYVCLNEY

HGAL1      TVKLPDGYEFKFPNRLN-LEAINYMAADGDFKIKCVAFD---------------------
HGAL2      KVKLPDGHELTFPNRLG-HSHLSYLSVRGGFNMSSFKLKE--------------------
HGAL3      KVAVNDAHLLQYNHRVKKLNEISKLGISGDIDLTSASYTMI-------------------
HGAL4-ND   KVVVNGNPFYEYGHRLP-LQMVTHLQVDGDLQLQSINFIGGQPLRPQGPPMMPPYPGPG-
HGAL4-CD   KVYANGQHLFDFAHRLSAFQRVDTLEIQGDVTLSYVQI----------------------
HGAL7      KAVVGDAQYHHFRHRLP-LARVRLVEVGGDVQLDSVRIF---------------------
HGAL8-ND   QVAVNGKHTLLYGHRIG-PEKIDTLGIYGKVNIHSIGFSFSSDLQSTQASSLEL-TEI--
HGAL8-CD   KVAVNGVHSLEYKHRFKELSSIDTLEINGDIHLLEVRSW---------------------
HGAL9-ND   KVMVNGILFVQYFHRVP-FHRVDTISVNGSVQLSYISFQNPRTVPVQPAFSTVPFSQPVC
HGAL9-CD   KVAVDGQHLFEYYHRLRNLPTINRLEVGGDIQLTHVQT----------------------
HGAL10     QVMVNGQSSYTFDHRIK-PEAVKMVQVWRDISLTKFNVSYLKR-----------------
HGAL12-ND  KVSVNGQHFLHFRYRLP-LSHVDTLGIFGDILVEAVGFLNINPFVEGSREY----PA---
HGAL12-CD  KLALNGQGLGATSMNQQALEQLRELRISGSVQLYCVHS----------------------
HGAL13     EIKVNGIRIYGFVHRIP-PSFVKMVQVSRDISLTSVCVCN--------------------
HAGL14     KVMVNGQRIYNFAHRFP-PASVKMLQVFRDISLTRVLISD--------------------
HGAL16     EVKVNGEYIYAFVHRIP-PSYVKMIQVWRDVSLDSVLVNNGRR-----------------
```

Figure S1. A) Structural types of galectins. Based on their structural organization, galectins are divided into proto, chimera, and tandem-repeat types. Prototype galectins are composed of one or two identical CRDs forming non-covalent homodimers; the chimera type, represented only by Gal-3, is composed by an N-terminal peptide bearing two phosphorylation sites, followed by nine collagen-like repeats and a C-terminal CRD; and tandem-repeat type galectins contain two different CRDs (N-CRD and C-CRD) covalently connected by a linker peptide. **B) Multiple amino acid sequence alignment of human galectin CRDs.** Sequences were obtained from UniprotKB (reviewed sequences) and aligned using ClustalOmega. N- and C-CRDs of tandem-repeat galectins were aligned independently. Conserved residues, conservative substitutions, and semi-conservative substitutions in at least 15 out of the 16 aligned sequences are highlighted in yellow, blue, and green, respectively.
